# Sandwich Generation Caregiving Among Baby Boomer and Generation X Caregivers in the United States

**DOI:** 10.1093/geroni/igab046.353

**Published:** 2021-12-17

**Authors:** Christina Miyawaki, Erin Bouldin, Eva Jeffers, Lisa McGuire

**Affiliations:** 1 University of Houston, Houston, Texas, United States; 2 Appalachian State University, Boone, North Carolina, United States; 3 Centers for Disease Control and Prevention (CDC), Atlanta, Georgia, United States; 4 CDC, Atlanta, Georgia, United States

## Abstract

Sandwich generation caregivers are generally those who care for both a child and an older adult. Baby Boomer and Generation X belong to this age cohort. Using data from the 2015-2018 Behavioral Risk Factor Surveillance System Caregiver Module, we compared the prevalence and characteristics of sandwich caregivers across these two generations. Data represent adults from 44 jurisdictions. We categorized caregivers into generations using their age at the time of the survey (N=34,777). Sandwich caregivers were classified as those who lived with a child (≤18 years) and provided care/assistance to a parent/grandparent with a long-term illness/disability during the past 30 days. Prevalence ratios (PR) from log-binomial regression models that included generation, sandwich caregiver status, sex, and race/ethnicity were used to compare weighted estimates. Six percent of Baby Boomers and 31% of Generation X were sandwich caregivers (p<0.001). In adjusted models, sandwich caregivers had a lower prevalence of any chronic health condition (PR=0.77, p=0.01) and fair/poor health (PR=0.87, p=0.003) than other caregivers, but similar frequent mental and physical distress prevalence. Baby Boomer caregivers were more likely to report a chronic health condition, fair/poor health, and frequent physical distress than their Generation X counterparts, but less likely to report frequent mental distress. Sandwich caregivers in these generations appear to be in better health than other caregivers. Nonetheless, it is critical to support the needs of sandwich caregivers as they age, given their important role in meeting the needs of both children and older adults and the additional challenges created by the pandemic.

